# Age and Spatial Peculiarities of Non-neoplastic Diseases of the Skin and Subcutaneous Tissue in Kazakhstan, 2003–2015

**Published:** 2017-11

**Authors:** Nurbek IGISSINOV, Dariyana KULMIRZAYEVA, Zarina BILYALOVA, Gulnur AKPOLATOVA, Marzya MAMYRBAYEVA, Galina ZHUMAGALIYEVA

**Affiliations:** 1. Central Asian Cancer Institute, Astana, Kazakhstan; 2. Dept. of General Pharmacology, Astana Medical University, Astana, Kazakhstan; 3. Dept. of General Practitioner Nº 1 and Communicative Skills, West-Kazakhstan Marat Ospanov State Medical University, Aktobe, Kazakhstan; 4. Dept. of Infectious Diseases and Children`s Infections, West-Kazakhstan Marat Ospanov State Medical University, Aktobe, Kazakhstan

**Keywords:** Incidence, Non-neoplastic diseases, Skin and subcutaneous tissue, Spatial analysis, Time trends

## Abstract

**Background:**

Arrangement of effective management aimed at improving dermatological services and consistent care of patients with skin diseases depends on understanding the epidemiological situation.

**Methods:**

This retrospective study presents an epidemiological assessment of non-neoplastic skin and subcutaneous tissue diseases in Kazakhstan registered in 2003–2015.

**Results:**

The yearly incidence rate of the diseases among the whole population was in average 3,341.8±121.1 per 100000 population. This represents 4835.0±156.1 for children, 5503.2±141.8 for adolescents and 2646.6±106.7 for adults per 100000 inhabitants. Space and time incidence rate was evaluated according to the administrative division. The overall trend decreased to 3.5% in children to 2.8% in adolescents to 1.9%, and in adults to 3.9%. Considerable variation in rates was seen across the country, with highest rates in East Kazakhstan, Mangystau and Aktobe regions, the lowest – in Atyrau and South-Kazakhstan regions.

**Conclusion:**

Non-neoplastic diseases of skin and subcutaneous tissue continue to be an urgent public health problem, especially among children in many regions of Kazakhstan.

## Introduction

The skin is one of the largest organs in the body with considerable role (1). It is not a simple and inert covering but a sensitive, dynamic interface between us, and the outside world (2) often subjected to aggressive external factors: such as mechanical, physical, chemical influence, as well as infectious agents (3–7). However, skin diseases caused by exogenous factors mostly diagnosed and treated much easier compared to diseases caused by endogenous factors, which usually include diseases of internal organs, especially liver, gastrointestinal, metabolic, endocrine, nervous, and lymphatic systems, causing lesion of the skin and mucous membranes (8).

Skin disease is an enormous health burden and the most frequent reason for people to consult their general practitioner. It can be found in all cultures, at all ages, both in men and women, and affects 30%–70% of individuals and even higher at-risk subpopulations (2, 9). For example, approximately 24% (12.9 million) of the population present to general practitioners with a skin problem each year in England and Wales (2). In the USA, 37.9 million visits were made to an office-based dermatologist for dermatological conditions in 2001. In Australia, 3.5% of hospital registration was for diseases of skin and subcutaneous tissue in 2001–2002 (10). Herewith specially collected data from four specialist dermatology departments in England showed that specialists most commonly see people with skin lesions (35%–45%), eczema, psoriasis and acne (2, 8). In USA psoriasis affects greater than 3% of the population, or more than 5 million adults (11) and about 15 million of people have eczema, of which 17% are children (12, 13). Globally in 2010, skin diseases were the 4th leading cause of nonfatal burden, expressed as years lost due to disability, and two individual skin conditions were in the top ten most prevalent diseases (fungal infection and acne vulgaris) (14).

In Kazakhstan, non-neoplastic skin and subcutaneous tissue diseases (SSTD) take the 5th place and constitute 6.08% among all diseases (15). The most common disease in all regions is eczema and psoriasis that is more prevailed in ecologically unfavourable regions (16). Kazakhstan is a very large country characterized by different environmental factors and living conditions. The arrangement of effective management aimed at improving dermatological services and consistent care of patients with skin diseases depends on understanding the epidemiological situation. Therefore, detailed study of epidemiological characteristics, geographical variations, changes in incidence of skin diseases will allow us to monitor its trends and further assess the impact of possible causal risk factors.

The aim of the present study was to conduct a total epidemiological analysis of non-neoplastic SSTD in Kazakhstan and assesses their changes over time, focusing on geographical area and age of population.

## Materials and Methods

The sources of information were the materials of state records of patients with firstly registered non-neoplastic SSTD (ICD–L00-L99). The data were extracted from the annual statistics reporting form of the Ministry of Healthcare and Social Development of the Republic of Kazakhstan for 2003–2015.

Data on the population density of different ages and geographical spread were obtained from the Statistics Committee of the Ministry of National Economy of the Republic of Kazakhstan (17). According to the law of the Republic of Kazakhstan “About State Statistics” (18), the information in the summary report is confidential and may only be used for statistical purposes. According to ICD, 10 diseases classification nonneoplastic skin and subcutaneous tissue diseases include more than 1000 different skin or skin-related illnesses: infections of the skin and subcutaneous tissue, bullous disorders, dermatitis and eczema, papulosquamous disorders, urticaria and erythema, diseases of the skin and subcutaneous tissue related to radiation exposure, diseases of the skin appendages and other diseases of the skin and subcutaneous tissue.

In this retrospective study, the incidence data were examined in separate groups: children under the age of 15, adolescents (15–17 yr old), and adults (18 and older) as well as the whole population in general. The materials were collected and analyzed by administrative-territorial division of the country (14 regions and 2 main cities: Astana and Almaty).

Various methods of biomedical statistics, such as extensive and intensive indexes, mean value, 95% confidence interval, average annual growth/decline rates (T, %) were used (19).

For the calculation of the average annual growth/decline rates of the dynamic series, the average geometrical was used, which is equal to:

$$T=\sqrt[{n}]{T_{1}\times T_{2}\times T_{3}\times T_{n}}$$

Where *T* is annual rate of growth/loss and *n* is number of rates.

In compiling the map were used the skin and subcutaneous tissue incidence rates for a 13-yr period (2003–2015). We used a method for compiling maps, proposed (20) in 1974, based on the definition of a standard deviation (*σ*) from an average of (*x*). The scale levels are calculated as follows: taking *σ* as an interval, we defined the maximum and minimum levels of disease according to this formula: *x±1.5σ*, with the minimum level of *x*−*1.5σ* and a maximum equal to *x+1.5σ*. Then we defined the scale levels of the map: *1) (x*−*1.5σ)+σ; 2) (x*−*1.5σ)+2σ; 3) (x*−*1.5σ)+3σ*, etc.; a grouping of indicators is derived from the formula *x±0.5σ*, corresponding with the average level *(x*−*0.5σ* and *x+0.5 σ)*; the values that are distant from the average incidence by σ, show lower *((x*−*0.5σ)*−*σ)* and higher *((x*−*0.5σ)+σ)* values. When grouping a parametrical series for construction of equal intervals was used, a formula proposed (21):

$$\gamma =\frac{X_{\max}–X_{\min}}{1+3,22\;\text{lg}\;n}$$

Where *X*_*max*_ is a maximum index; *X*_*min*_ is a minimum index; and *n* is a number of population, i.e. amount of areas and cities.

Data collection and analysis of the materials were made using Microsoft Excel computing program and BIOSTAT, EpiInfo 7.

## Results

### Overall and Age Dependent Incidence Rates Over Time

**Fig. 1:**
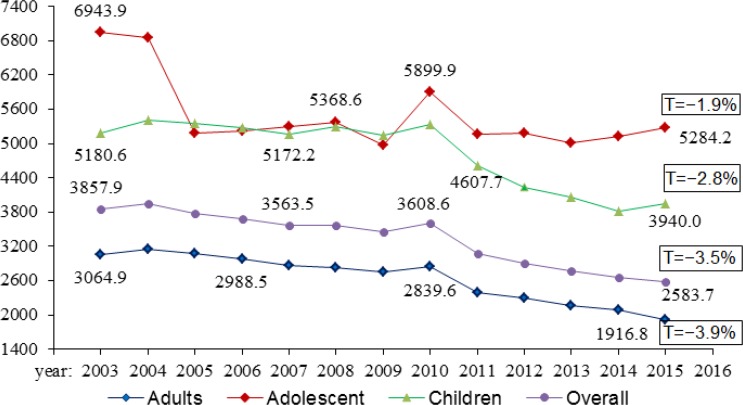
Dynamics of incidence rates of non-neoplastic skin diseases in Kazakhstan for 2003–2015

In the whole country, the total number of all registered patients with skin diseases, for the reported years, amounted to 6897726. Out of total registered cases 2478522 in the pediatric population, represent 35.9%. There were 612546 cases registered for adolescents, which are 8.9%, and majority of cases were among adults - 3806658 which is 55.2% of total population. Despite the fact that more than half of disease cases were reported in adult population, the average incidence rate of non-neoplastic skin illnesses per 100000 populations for 2003–2015 showed that skin problems most often were seen in children and adolescents: 4835.0±156.1^0^/_0000_ and 5503.2±141.8^0^/_0000_, respectively. In adult population, the average incidence rate was 2646.6±106.7^0^/_0000_ (Fig. 1).

The decrease in incidence rates over time in all age groups was noted, more prominently in adults. The overall trends decreased to 3.5%, in children to 2.8%, in adolescents to 1.9%, and in adults to 3.9%. In 2010, a sudden increase of non-neoplastic SSTD in all age groups was observed.

### Geographical Variation

**Fig. 2:**
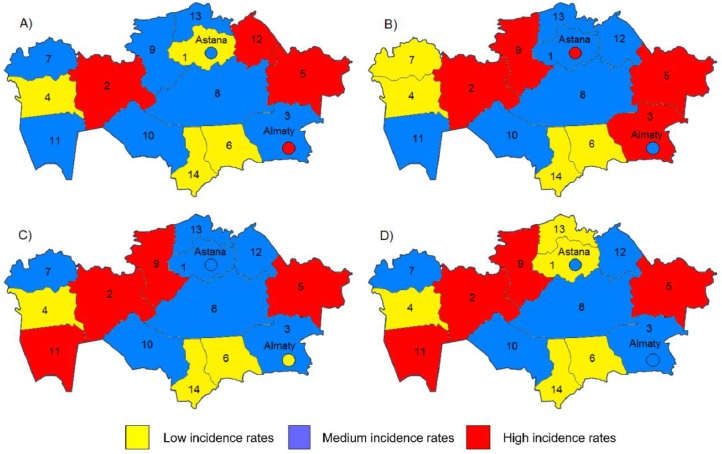
Distribution of skin and subcutaneous tissue diseases average incidence rate in Kazakhstan for 2003–2015 on map: a) children, b) adolescents c) adults and d) overall

The regional features of skin disease distribution according to the age groups and overall are shown in Table 1 and on map of the Kazakhstan (Fig. 2).

**Table 1:** The regional features of skin disease distribution according to the age groups

***Region***	***Overall***		***Children under 15 yr***		***Adolescents 15–17 yr***		***Adults (18 yr and older)***	
	**Incidence, ^0^/_0000_ P±m**	**T, %**	**Incidence, ^0^/_0000_ P±m**	**T, %**	**Incidence, ^0^/_0000_ P±m**	**T, %**	**Incidence, ^0^/_0000_ P±m**	**T, %**
Atyrau	1787.3±139.5	−6.5	2378.6±156.6	−5.3	1407.9±74.7	1.3	1570.5±158.6	−8.1
South Kazakhstan	2315.1±59.2	−1.3	2967.6±88.3	−1.3	2838.4±79.8	−0.8	1898.0±48.3	−1.4
Akmola	2811.7±136.8	−5.0	3775.9±165.8	−4.1	4461.2±261.9	−5.8	2383.0±117.3	−4.9
Zhambyl	2814.6±83.1	−2.6	4168.4±89.3	−0.4	4024.4±193.1	−3.5	2089.7±97.7	−4.6
North Kazakhstan	2920.6±69.2	+0.9	4584.4±190.6	+2.9	4395.9±175.3	+1.4	2391.0±55.7	+0.2
Karaganda	3314.7±194.1	−5.7	4730.6±278.7	−3.8	4939.1±249.8	−3.6	2800.4±184.6	−6.7
Republic	3341.8±121.1	−3.5	4835.0±156.1	−2.8	5503.2±141.8	−1.9	2646.6±106.7	−3.9
West Kazakhstan	3349.5±111.4	−2.5	5129.8±297.0	−4.4	3925.3±209.3	+2.2	2737.3±94.4	−1.6
Pavlodar	3377.6±69.6	−0.8	5904.0±152.6	+2.4	6425.7±221.6	+3.1	2513.5±108.1	−2.8
Almaty city	3393.3±129.1	−3.7	8127.9±383.1	−4.6	5289.5±250.6	−4.5	2131.1±65.4	−2.6
Astana city	3447.1±127.7	−0.4	5526.0±264.5	−3.6	6999.7±449.6	−4.0	2736.4±116.2	+1.2
Kyzylorda	3539.3±305.4	−8.4	4438.3±231.0	−4.4	4975.6±412.6	−7.2	2950.9±324.4	−11.8
Almaty	3699.9±276.3	−7.1	4784.5±363.4	−6.2	10067.8±934.2	−2.6	2775.2±220.3	−7.7
Kostanai	3801.6±130.6	−2.2	5442.8±114.9	−1.6	7081.8±181.2	+0.5	3167.4±130.5	−2.2
Aktobe	4349.6±133.3	−3.0	6512.9±259.3	−3.9	7720.1±113.4	+0.5	3304.0±84.8	−2.4
Mangystau	4639.9±221.5	−4.0	5718.2±196.6	−2.0	4509.0±306.8	+0.5	4146.5±274.5	−5.9
East Kazakhstan	4676.6±124.8	−2.6	7626.2±138.4	−1.3	8331.9±114.8	+1.2	3654.8±128.4	−3.4

According to territorial analysis, all nonneoplastic SSTD incidences rates were grouped into three categories: regions with low, medium and high incidence rates (per 100000 inhabitants). The highest average incidence rates were found in East Kazakhstan, Mangystau and Aktobe regions, the lowest – in Atyrau and South-Kazakhstan regions. In the dynamics the most expressed decline rates were found in Atyrau (T= −6.5%), Almaty (T= −7.1%) and Kyzylorda regions (T= −8.4%). In Pavlodar and North-Kazakhstan regions increase of the incidence rates were marked.

## Discussion

Skin diseases are among the most prevalent health problems worldwide. The most non-fatal skin disease burden per 100000 populations in 1990–2010 experienced such countries as Singapore, Brunei, Sudan and Hungary, while the least burden was found in Albania, Lithuania, Romania and Indonesia (14). In Kazakhstan, the average incidence rate of SSTD was 3341.8±121.1 per 100000 populations. However, the real incidence rates may be much higher, taking into account that the majority of the population do not seek medical help or prefer home and self-treatment (8).

One of the possible causes of skin problems is ecological and climatic factors. For example, in tropical resource-poor regions (Oceania, sub-Sahara Africa, southeast Asia, and the tropical Americans) skin infections assume a higher proportion of the disease burden compared to temperate regions (14, 22, 23). Kazakhstan is a big country characterized by different ecological, environmental and living conditions. The highest average incidence rates were found in East Kazakhstan, Mangystau and Aktobe regions, the lowest – in Atyrau and South-Kazakhstan regions. East Kazakhstan region is well known all over the world for the Semipalatinsk nuclear testing site located on its territory. Between 1949 and 1989, 456 nuclear tests (22) had extreme effect on the local environment. Therefore, children with genetic diseases, leukemia, various skin diseases and cancer are common here. In 1991, the site was closed, but lots of population still has health problems (24, 25).

One of the probable causes of low levels of skin diseases in the South Kazakhstan region is the use of a population of large quantities of grapes which are a considered a strong natural antioxidant. Resveratrol and other grape compounds, as well as whole-grape products, have shown significant assurance in health promotion and disease management, One of them protection against ultraviolet radiation and anti-inflammatory properties (26, 27). In addition, most of the population of the southern region usually tries to do their business in the morning or evening times, when solar activity is not so strong or already reduced. All these assumptions point to the need for further detailed research studies focusing on etiological, ecological factors of non-neoplastic SSTD on regional levels.

Another important issue on which we must focus is that skin problems once appeared in childhood can further cause disability with high levels seen in teenage years and in senility. For instance, most of the chronic skin conditions, such as atopic eczema, psoriasis, and vitiligo are not immediately life threatening but are recognized as a considerable burden on health status and quality of life, including physical, emotional and financial consequences (2, 28). Herewith, skin diseases most frequently cause mental suffering of patients, because unlike other diseases, have external manifestations. Therefore, there is no doubt that skin problems are one of the largest burdens worldwide and a source of considerable loss of healthy life and, thus, must be of great importance for practical and public health. Unfortunately, the method of data collection in Kazakhstan does not allow us to get information more detailed regarding division the skin diseases into infectious and non-infectious, or etiological factors. Therefore, it is very difficult to compare our results to those of different countries. We will try to overcome this problem in future researchers. We hope the present study will contribute to the development of dermatological diagnostic more effective and therapeutic interventions and preventive strategies in each region, making an accent to the most vulnerable regions and will serve as the basis for future studies of the skin and subcutaneous tissue diseases devoted to determine the causative factors locally and in general.

## Limitation of the study

Although this research was carefully prepared, there were some unavoidable limitations and shortcomings. First, due to the lack of information in the reporting forms of the Ministry of Healthcare and Social Development of the Republic of Kazakhstan concerning SSTD we cannot split the diseases of skin and diseases of sub-cutaneous tissue, as well as we cannot identify the type of disease in that reports. Second, there is no information about the sex differentiation of SSTD. Third, the reporting forms do not provide the information about the diagnosis of the diseases.

## Conclusion

This is the first study conducted in Kazakhstan to provide detailed information, including the common and regional characteristics of nonneoplastic SSTD over an extended period. The results showed a decrease of skin illnesses in all age groups in Kazakhstan, but they continue to be an urgent public health problem, especially among children in many regions of the country.

## Ethical considerations

Ethical issues (Including plagiarism, Informed Consent, misconduct, data fabrication and/or falsification, double publication and/or submission, redundancy, etc) have been completely observed by the authors.
